# Protein Extractions from *Amphistegina lessonii*: Protocol Development and Optimization

**DOI:** 10.3390/life11050418

**Published:** 2021-05-05

**Authors:** Michele Betti, Caterina Ciacci, Sigal Abramovich, Fabrizio Frontalini

**Affiliations:** 1 Department of Biomolecular Science, Urbino University, 61029 Urbino, Italy; michele.betti@uniurb.it (M.B.); caterina.ciacci@uniurb.it (C.C.); 2 Department of Earth and Environmental Sciences, Ben Gurion University of the Negev, Beer Sheva 84105, Israel; sigalabr@bgu.ac.il; 3 Department of Pure and Applied Sciences, Urbino University, 61029 Urbino, Italy

**Keywords:** protein, protocol, SDS-PAGE, benthic foraminifera, *Amphistegina*

## Abstract

Proteins are essential to life, and the evaluation of their content, identification, and modification represents a fundamental assay in biochemistry research. Different analytical techniques and protocols have been specifically designed but have rarely been compared. Here, we test and compare a variety of methodologies and treatments for the quantification of proteins in *Amphistegina lessonii*, a larger symbiont-bearing benthic foraminiferal species. These analyses specifically include (a) lysis buffer (homemade vs. RIPA), (b) protein assays (Lowry, BCA, and Bradford), (c) ultrasonic bath treatment, and (d) protein staining (silver staining vs. Coomassie blue). On the basis of the comparative outcome, we suggest using the homemade lysis buffer, Lowry or BCA assays, ultrasonic bath treatment, and silver stain to maximize the extraction and characterization of protein for *A. lessonii*. This protocol might be suitable and extended to other benthic foraminiferal species, including the smaller ones.

## 1. Introduction

The recent advances of “omic” technologies (i.e., genomics, proteomics, metabolomics, and transcriptomics) have primarily provided fundamental information about targeted biological samples and opened new research areas [1]. The application of these “omics” allows the detection of biological variations at the molecular functional levels [2]. The implementation of such technologies has stimulated the development of protocols, typically target-specific, and new techniques that require being intercalibrated to achieve comparable results.

Proteins are essential to life because they are functionally involved in a vast array of cellular activities from energy production to metabolism through DNA replication. The evaluation of protein content, its identification, and modification represent a fundamental assay in biochemistry researches that occasionally remains challenging [3]. In this context, several analytical techniques and protocols specifically designed for purification, protein extraction, quantification, and identification have been suggested [4]. These approaches span from ultraviolet absorption spectroscopy to the more standard dye-based colorimetric measurements using Lowry and Bradford assays, among others [4], and all have pros and cons. Assay choice is carefully considered and is mainly related to the targeted biological samples, sample volume, recovery, protein aggregation, and chemical reactions [4], as well as the objective and applications.

Phytoplankton, zooplankton, and benthic organisms are among marine groups that have been targeted for protein content analyses, although using different and sometimes incomparable techniques [5]. Protein analysis has recently found a new application area in the biomonitoring (i.e., pollution) or evaluation of climatic change effects (e.g., warming, acidification). In this context, benthic foraminifera—single-celled organisms— have been extensively used as bioindicators in environmental biomonitoring from local to global scales and, more recently, as ecotoxicological bioassays [6,7,8,9]. The investigation of physiological and biochemical changes on foraminifera ascribed to various stressing factors such as heavy metals, organic matter, and thermal pollution (i.e., global climate changes) can provide valuable information on the marine environment’s environmental quality. Unfortunately, to the best of our knowledge, only a few investigations have been performed to characterize the protein content of foraminifera [10,11,12,13,14,15]. Doo et al. [10] provided a measurement of the total protein content (i.e., 1.95 ± 0.04 µg/individual) in a foraminiferal species (i.e., *Baculogypsina sphaerulata*). The protein yield was documented in the smaller benthic foraminiferal species *Ammonia tepida* (0.066 ± 0.012 µg/individual), *Ammonia beccarii,* (0.085 µg/individual), *Elphidium crispum* (0.04 µg/individuals), and *Massilina secans* (0.231 ± 0.34 µg/individual) [12]. The protein content of selected larger benthic foraminiferal (LBF) species, namely *Amphisorus hemprichii* (77.55 ± 2.79 µg/individual), *Marginopora vertebralis* (171.28 ± 1.37 µg/individual), and *Calcarina gaudichaudii* (from 16.34 ± 0.46 to 16.34 ± 0.46 µg/individual) was reported by Doo et al. [11]. The same authors also provided an accurate description of the protein protocol specifically designed for foraminifera. The determination protocol details the protein extraction, electrophoresis, and western blotting. Changes in the proteome composition in *Amphistegina gibbosa*—a diatom-bearing benthic foraminifera—when thermally stressed were evaluated by Stuhr et al. [15].

The methodologies used for protein assay in foraminifera have been inherited from traditional methods. However, the wide variety of such methods from extraction to staining makes it rather difficult to compare the results [5]. Indeed, no comparative tests on the reliability and performance of the different assays have been performed on foraminifera so far. Hence, the objective of the present study is to apply and compare the outcomes of different methods of lysis, protein assays, and protein staining on foraminifera. *Amphistegina lessonii*—a symbiont-bearing foraminiferal species—was chosen as the model organism.

## 2. Materials and Methods

### 2.1. Individual Collection

Individual *A. lessonii* (Amphisteginidae, Foraminifera) were collected from pieces of rock pebbles within the Gulf of Aqaba-Eilat (Red Sea, Israel) in October 2020. Pebbles were collected at water depths of 1 to 2 m in a reference site that is considered clean and has been used for previous investigations [8]. Once in the laboratory, the pebbles were immediately scrubbed using a small brush into a bucket containing seawater from the collection site for separation living *A. lessonii* individuals. Adult living individuals (600 to 800 µm) were placed in 50 mL tubes and delivered to Urbino University (Italy). Once in the laboratory, individuals were placed in 100 mm glass Petri dishes with water at the sampling site for acclimatization for 2 weeks at 25 °C and 12:12 light and dark cycles. Living individuals showing a golden-brown color and exhibiting pseudopodial activity were then picked under a stereomicroscope for specific tests to develop the protocol. Three replicated batches containing 40 individuals of *A. lessonii* were ground with a Teflon needle, homogenized, and mixed for each treatment (i.e., lysis buffer, protein assay, ultrasonic bath treatment, and protein stain).

### 2.2. Lysis

Batches were ground and homogenized with two different lysis buffers—a) homemade lysis buffer and b) RIPA buffer. For the homemade lysis buffer, samples were lysed with 0.5 mL of ice-cold lysis buffer [50 mM Tris·HCl, pH 7.8, 0.25 M sucrose, 1% (wt/vol) SDS, 1 μg/ml pepstatin, 10 μg/ml leupeptin, 2 mM sodium orthovanadate, 10 mM NaF, 5 mM EDTA, 5 mM N-ethylmaleimide, 40 μg/ml PMSF, and 0.1% Nonidet P-40] and sonicated for 45 s at 100 W.

For RIPA buffer, samples were lysed with 0.5 mL of ice-cold RIPA complete lysis buffer and sonicated for 45 s at 100 W. The homogenate was divided into two aliquots, one of which was placed for 10 min in an ultrasonic bath. Samples were boiled for 10 min and then centrifuged for 20 min at 14,000× *g* to remove insoluble debris, and supernatants were recovered. Then, the homogenate was divided into two aliquots, one of which was placed for 10 min in an ultrasonic bath, whereas the second one was not sonicated.

### 2.3. Protein Assays

Three methods were considered and compared to determine the total protein content:

1. Lowry’s method [16].

Mixing 25 μL of sample with 2375 μL solution A (dH_2_O, Na_2_CO_3_ 1M, NaOH 0.25M, Na-K tartrate 0.2%, CuSO_4_ 0.1%) and incubating for 10 min, adding 1:4 (*v*/*v*) Folin reagent, mixing, incubating for 10 min in darkness, and reading the absorbance at 700 nm.

2. Bradford’s method [17].

20 μL of sample was mixed with 1 mL of solution B (dH_2_O and Bradford reagent), and then the absorbance was read at 595 nm.

3. BCA method [18].

Mixing 25 μL of sample with 500 μL of working reagents (50 parts of BCA reagent A with 1 part of BCA reagent B from Pierce BCA protein assay kit), incubating for 2 h and reading the absorbance at 565 nm.

Blanks (i.e., buffered solutions without *A. lessonii* individuals) were also carried out.

### 2.4. Statistical Analysis

Differences in mean values of protein content among treatments (i.e., lysis buffer, ultrasound, and assay) were analyzed by one-way analysis of variance (ANOVA). Data were square-root transformed to meet ANOVA assumptions. Post-hoc analysis was performed using Tukey’s honestly significant difference (HSD) tests. The significance level adopted was 95% (α = 0.05).

### 2.5. SDS-PAGE

Gel electrophoresis was carried out on polyacrylamide (with an acrylamide/Bis ratio of 30:1) slab gels (60 × 80 × 1 mm^3^) using the discontinuous buffer system of Laemmli. The separating gel of 10% polyacrylamide was underlain with 4% stacking gel, and the running buffer consisted of 0.025 M Tris, 0.2 M glycine, and 0.1% SDS. Gels were run in a Mini-Protein II dual slab cell (Bio-Rad) at a constant current of 10 mA per slab gel using a Power PAC 300 (Bio-Rad). Samples were mixed 1:1 (vol/vol) with sample buffer (0.5 M Tris·HCl, pH 6.8, 2% SDS, 10% glycerol, 4% 2-mercaptoethanol, and 0.05% bromophenol blue), and the samples (normalized for protein content before they were loaded to 20 μg of protein) were resolved.

### 2.6. Protein Staining

All working solutions for staining were prepared just before their use, and all steps were carried out at room temperature with shaking.

1. Silver Stain

This staining method was essentially according to [19]. For staining with silver, proteins were fixed in 40% methanol/10% acetic acid (*v*/*v*) for 30 min and in 10% ethanol/5% acetic acid (*v*/*v*) for 30 min and then oxidized for 5 min in a Bio-Rad Oxidizer (1:10 in dH_2_O), washed in dH_2_O for 15 min, incubated with silver reagent (1:10 in dH_2_O) for 20 min, washed in dH_2_O for 1 min, developed for 10 min with Developer Bio-Rad (32 g of developer per L of deionized water), and stopped in 5% acetic acid (*v*/*v*).

2. Coomassie Stain

Coomassie brilliant blue R250, an anionic dye, is the most popular stain for detecting proteins resolved in SDS-PAGE gels. The stain was prepared by dissolving 0.12% (*w*/*v*) dye in 50% (*v*/*v*) methanol and 10% (*v*/*v*) acetic acid. SDS-PAGE gels were stained for 2 h and de-stained with two changes every 2 h of 20% (*v*/*v*) methanol and 10% (*v*/*v*) acetic acid.

## 3. Results

**Figure 1:**
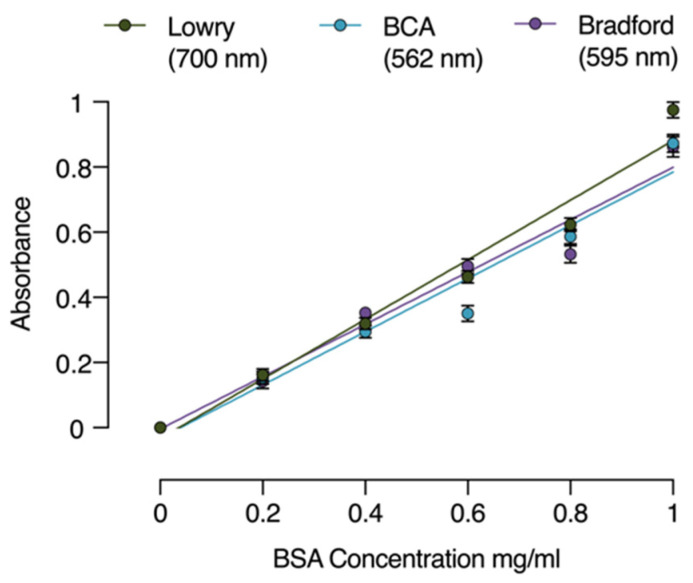
Standard curves: absorbance vs. protein content based on a BSA range of 0 to 1 mg/mL for different considered protein assays (i.e., Lowry, BCA, and Bradford). Data are expressed as mean ± standard deviation.

Figure 1 shows the standard curves related to the three protein assays (i.e., Lowry, BCA, and Bradford) using a series of bovine serum albumin (BSA) standards in the 0 to 1 mg/mL range. The absorbance was measured at 700 (Lowry), 562 (BCA), and 595 nm (Bradford) and plotted against BSA. Linearity was checked with a linear regression analysis; the correlation coefficient and calibration curve equation were then calculated (Table 1).

**Figure 2:**
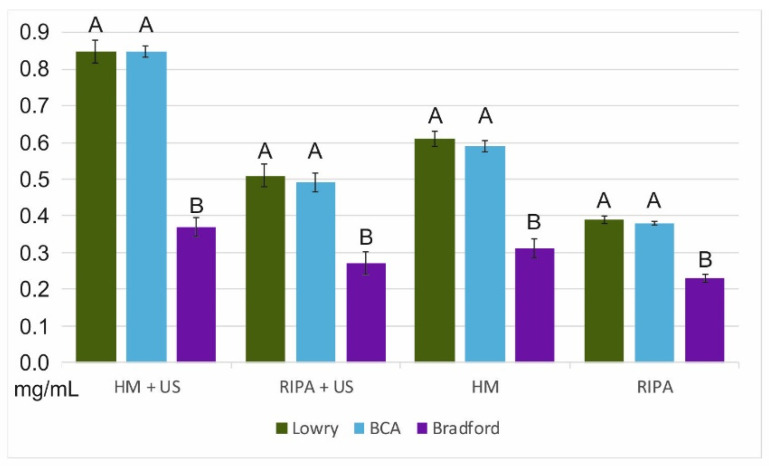
Comparison of protein content (mg/mL) per different treatments with and without an ultrasonic bath treatment (US): lysis buffer: homemade (HM) and RIPA; assays: Lowry, BCA, and Bradford. Data are expressed as mean ± standard deviation. Letters denote significant differences (Tukey’s HSD post-hoc test) between methods for each treatment (homemade lysis buffer + ultrasonic bath treatment, RIPA lysis buffer + ultrasonic bath treatment, homemade lysis buffer, and RIPA).

The protein yield ranged from 0.23 to 0.85 mg/mL (Figure 2; Table 2). Marked variations in the protein yield were associated with the different considered methodologies, namely (a) lysis buffer, (b) protein assays, and (c) ultrasonic bath treatment (Figure 2; Table 2).

The homemade lysis buffer extracts from 0.31 ± 0.05 to 0.85 mg/mL ± 0.06 (0.597 mg/mL, on average) were comparatively and significantly (F_1,34_ = 14.89, *p* = 0.0005) higher than the RIPA (0.23 ± 0.02–0.51 ± 0.06 mg/mL; 0.378 mg/mL, on average). The highest yield was found with the Lowry methods (0.39 ± 0.02–0.85 ± 0.06 mg/mL; 0.59 mg/mL, on average), although the BCA method provided a very similar but slightly lower yield (0.38 ± 0.01–0.85 ± 0.03 mg/mL; 0.58 mg/mL on average) (Figure 2; Table 2). On the other hand, the Bradford method consistently led to a much lower yield (0.23 ± 0.02–0.37 ± 0.05 mg/mL; 0.295 mg/mL on average) (Figure 2; Table 2). 

There was a significant assay effect on protein yield (F_2,33_ = 13.718, *p* = 0.00005). Tukey’s HSD post hoc test revealed a significant decrease in protein yield with the Bradford method (*p* < 0.01) compared to the Lowry and BCA methods, but no difference was found between these two latter methods. The homogenates placed in ultrasonic bath had a significantly (F_1,34_ = 4.36, *p* = 0.04) higher yield (0.27 ± 0.06–0.85 ± 0.06 mg/mL; 0.557 mg/mL on average) than those without it (0.23 ± 0.02–0.61 ± 0.04 mg/mL; 0.418 mg/mL on average).

**Figure 3:**
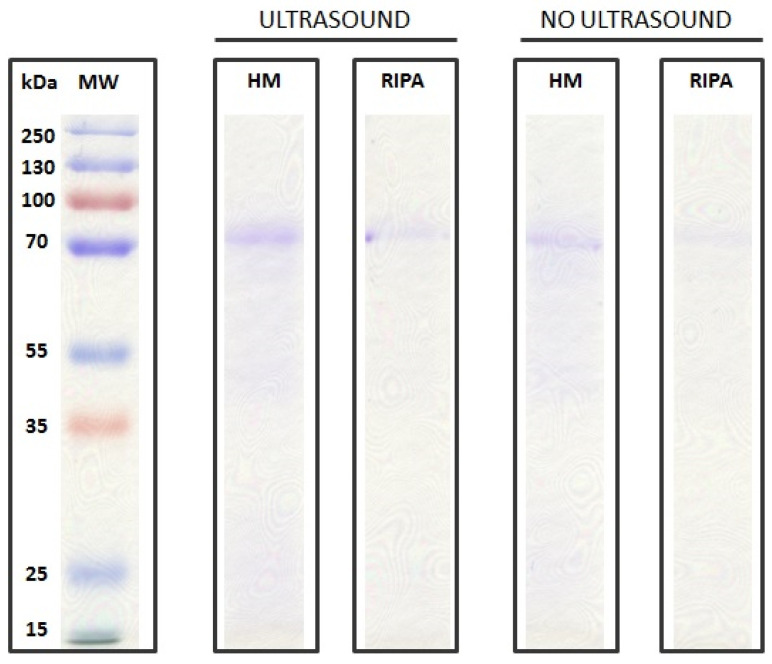
SDS PAGE acrylamide gel (12%) Coomassie blue-stained comparison among lysis buffers (homemade vs. RIPA) and ultrasonic bath treatment (20 µg of sample were loaded into each well). The SMW (standard molecular weight) is also reported.

**Figure 4:**
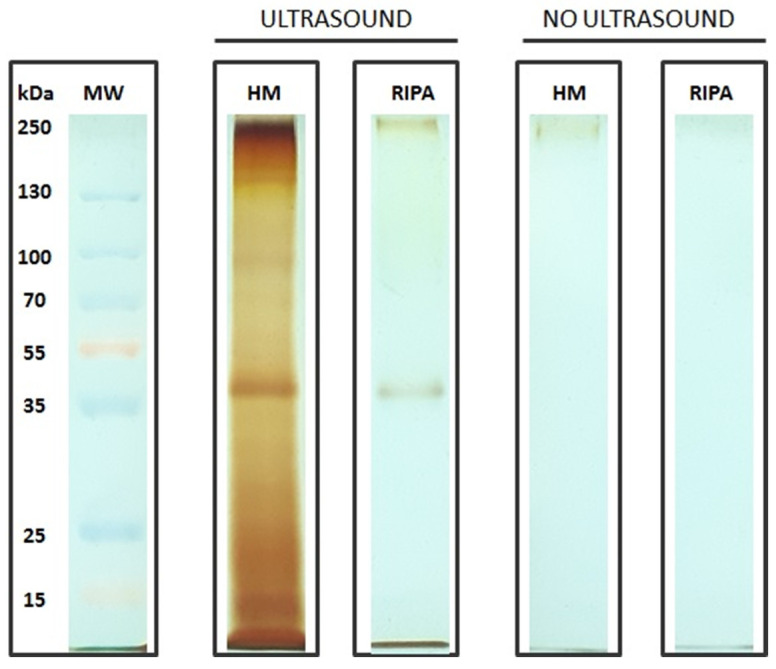
SDS PAGE acrylamide gel (10%) silver stain comparison among lysis buffers (homemade vs. RIPA) and ultrasonic bath treatment (5 µg of sample were loaded into each well). The SMW (standard molecular weight) is also reported.

On the basis of the different methodologies, the highest yield was obtained with the homemade lysis buffer, Lowry assay, and ultrasonic bath treatment which, combined, led to a total protein content of 6.1 µg per individual, which is slightly higher than the one with the BCA assay (6 µg per individual). The Coomassie blue stain (Figure 3) appeared to be less sensitive than the silver stain (Figure 4). This lower sensitivity was found whether or not the ultrasonic bath treatment was used.

## 4. Discussion

### 4.1. Comparison among Methodologies for Protein Assays in Large Benthic Foraminifera 

Different techniques for breaking the foraminiferal cellular membranes have been previously considered and include mechanically grinding with tweezers or a plastic pestle [10,11,12,15] and crushing to a fine powder with a mortar and pestle [14]. All these techniques involve the destruction of the foraminiferal tests. Alternatively, osmotic shock (with Milli-Q water and micro-filtered tap water), ultrasound (for 2 and 5 s), and NaOH treatments were proposed as methods to ensure the foraminiferal test’s preservation [13]. The same authors detailed the best technique following foraminiferal test characteristics (i.e., dimension of the apertures, foramens, and test robustness). On the basis of our goal of comparing the performance of the different protein assays, we used a destructive technique based on foraminiferal crushing with a Teflon needle.

The Thermo Scientific™ RIPA buffer is one of the most reliable buffers for lysing cultured mammalian cells from both plated cells and cells pelleted from suspension cultures. This buffer enables extraction of the cytoplasmic, membrane, and nuclear proteins and is compatible with many applications, including reporter assays, protein assays, immunoassays, and protein purification. This buffer has already been applied to lyse LBFs [10,11]. Other lysis buffers (i.e., 1) 80 mM potassium acetate, 5 mM magnesium acetate, 20 mM Hepes pH 7.5 by Heinz et al. [12], 2) 50 mM Tris-HCl (pH 7.8), 150 mM NaCl, 1% SDS and Complete Mini by Stuhr et al. [15], and 3) 100 mM Tris–HCl (pH 7.4) by Sabbatini et al. [14]) have been previously used on foraminifera. The here-used homemade lysis buffer has been developed over time by one co-author (MB) by modifying the already-existing protein extraction buffer to optimize protein extraction in marine organisms such as mussels, clams, and oysters. A substantially higher yield is associated with the homemade lysis buffer (0.597 mg/mL, on average) than the RIPA (0.378 mg/mL, on average). The homemade lysis buffer could, therefore, be used for accurate protein extraction in benthic foraminifera (i.e., *Amphistegina*).

No marked differences in protein yield were found between the Lowry and BCA methods, whereas the Bradford dosage led to a ~50% lower yield. Indeed, the Lowry and BCA methods also matched well with respect to measured protein and the standard serial dilutions, whereas the Bradford method underestimated all concentrations. The Lowry, BCA, and Bradford methods were all sensitive to microgram-level concentrations of protein, essential when only a limited amount of biological sample is available. These differences in protein detection ability among the three methods are ascribed to an overall underestimation of protein by the Bradford assay [20]. This underestimation by the Bradford method could be explained by its higher sensitivity to BSA than to a diverse pool of proteins [21]. Indeed, the Bradford dye reagent reacts primarily with arginine residues and does not equally detect all proteins leading, therefore, to this protein’s underestimation [22]. Of the three assays here discussed, the Lowry and BCA methods would be preferable over Bradford for invertebrate protein assays because it provides higher estimates of total protein and is not as affected by protein composition [23]. Both Lowry and BCA methods may, however, be insensitive to small peptides and amino acids [24]. The Lowry and BCA methods are, therefore, the most accurate protein measurement approaches in benthic foraminifera (i.e., *Amphistegina*). These findings are consistent with the outcomes of Martínez et al. [5] that among all seven tested methodologies involving colorimetric protein assays (i.e., Rutter, Rutter-SDS, Markwell, BCA, microBCA, Bradford, and microBradford) on different sizes of marine plankton, a mysid, and a jellyfish species, determined the BRAD and mBRAD as methods consistently underestimating protein. The same results were obtained in a wide range of marine organisms, from bacteria to algae [25,26]. To date, protein content has been quantified on foraminifera by using the Pierce^®^ BCA [10,11] and Bradford [12] assays, amino acid analysis [15], as well as copper and BCA solutions [13].

Significant differences were also associated with the use of the ultrasonic bath treatment (0.557 mg/mL, on average) compared to those without it (0.418 mg/mL, on average) (Figure 2, Figure 3). The capability of the ultrasonic bath to extract a higher content (ca. 26.4%) of low-abundance protein has already been pointed out [27]. Moreover, ultra-sonication has been documented to provide five-fold higher protein yields as compared to high-pressure homogenization [28]. Ultrasound treatment promotes the formation of voids (i.e., cavitation bubbles), thereby enhancing solid–liquid extraction [29]. The application of an ultrasonic bath on foraminifera is here considered a key approach to maximize protein extraction. This ability can be ascribed to the foraminiferal test, where the cell adheres to. Differences were also found in the considered staining gels (i.e., Coomassie blue vs. silver stain). The silver stain has been widely documented to be significantly more sensitive than Coomassie blue. Despite Coomassie blue being the most widely used gel, it can commonly detect 50 ng protein bands, whereas the silver stain has a sensitivity of 10 to 100 fold [30]. Different protein staining approaches have been used on foraminifera, including SYPRO^®^ Ruby (Invitrogen) [10,11], a staining solution (1 mM 4-chloro(1)naphthol and 0.015% H_2_O_2_ in 30 mM Tris pH 8.5 containing 6% methanol) [12], and silver nitrate [14].

### 4.2. Developing and Detailing a Protocol for Protein Assays

#### 4.2.1. Pre-Treatment

1. Preparations of all working reagents, solutions, and buffers.
2. Accurate selection of living foraminifera based on the presence of pseudopodial activity.
3. Clean foraminiferal tests with a brush and filtered seawater to remove all particles over the test.

#### 4.2.2. Extraction Buffer

1. Homogenization of LBFs by mechanically crushing foraminiferal tests until pulverized with Teflon in 1.5 mL Eppendorf vials containing 0.5 mL ice-cold homemade lysis buffer [50 mM Tris·HCl, pH 7.8, 0.25 M sucrose, 1% (wt/vol) SDS, 1 μg/mL pepstatin, 10 μg/ml leupeptin, 2 mM sodium orthovanadate, 10 mM NaF, 5 mM EDTA, 5 mM N-ethylmaleimide, 40 μg/mL PMSF, and 0.1% Nonidet P-40]. All manipulations are performed on ice to prevent protein degradation.
2. Sonication for 45 s at 100 W in ice.
3. Boil samples for 10 min.
4. Centrifuge for 20 min at 14,000× *g* to remove insoluble debris; supernatants were recovered.
5. Transfer the supernatant into a new 1.5 mL tube and preserve it at −80 °C.

#### 4.2.3. Protein Assay

1. Defrost the supernatant.
2. Prepare the standards (i.e., bovine serum albumin (BSA) protein standards in a solution of 1 mg/mL of H_2_O) only).
3. Sample preparation

(a) Lowry’s method

(a1) Mix 25 μL of sample with 2375 μL of solution A (dH_2_O, Na_2_CO_3_ 1M, NaOH 0.25M, Na-K tartrate 0.2%, CuSO_4_ 0.1%) and incubate for 10 min.

(a2) Add 1:4 (*v*/*v*) Folin reagent, mix, and incubate for 10 min in darkness.

(a3) Read the absorbance with a spectrophotometer at 700 nm.

(a4) Standard curve between BSA and absorbance at 700 nm.

(a5) Determine the protein concentrations from absorbance values. 

(b) Bradford’s method (Bradford, 1976) [17]

(b1) Mix 20 μL of sample with 1 mL of solution B (dH_2_O and Bradford reagent).

(b2) Read the absorbance with a spectrophotometer at 565 nm.

(b3) Standard curve between BSA and absorbance at 565 nm 

(b4) Determine the protein concentrations from absorbance values. 

#### 4.2.4. SDS-PAGE

1. Perform gel electrophoresis on polyacrylamide (with an acrylamide/Bis ratio of 30:1) slab gels (60 × 80 × 1 mm^3^) using the discontinuous buffer system of Laemmli.
2. The separating gel of 10% polyacrylamide is underlain with 4% stacking gel, and the running buffer consisted of 0.025 M Tris, 0.2 M glycine, and 0.1% SDS.
3. Run gels on a Mini-Protein II dual slab cell (Bio-Rad) at a constant current of 10 mA per slab gel using a Power PAC 300 (Bio-Rad).
4. Mix samples 1:1 (vol/vol) with sample buffer (0.5 M Tris·HCl, pH 6.8, 2% SDS, 10% glycerol, 4% 2-mercaptoethanol, and 0.05% bromophenol blue).
5. Normalize samples for protein content before loading to 20 μg of protein.
6. Boil all samples with sample buffer for 5 min to denature proteins.

#### 4.2.5. Silver Stain

1. Prepare protein staining just before use.
2. All steps are at room temperature with shaking with clean glass materials.
3. Fix protein in 40% methanol/10% acetic acid (*v*/*v*) for 30 min and in 10% ethanol/5% acetic acid (*v*/*v*) for 30 min.
4. Oxidize for 5 min in a Bio-Rad Oxidizer (1:10 in dH_2_O).
5. Wash in dH_2_O for 15 min and incubate with Silver reagent (1:10 in dH_2_O) for 20 min.
6. Wash in dH_2_O for 1 min and develop for 10 min with Developer Bio-Rad (32 grams of developer per liter of deionized water) and stop in 5% acetic acid (*v*/*v*).

### 4.3. Chemicals

Coomassie brilliant blue R250, sodium dodecyl sulfate (SDS), bromophenol blue, N′,N′,N′,N′-tetramethylethylenediamine (TEMED), β-mercaptoethanol, disodium ethylenediaminetetraacetic acid (Na_2_EDTA), Tris·HCl, sucrose, pepstatin, leupeptin, sodium orthovanadate, NaF, N-ethylmaleimide, PMSF, Nonidet P-40, Folin–Ciocalteu, Na_2_CO_3_, NaOH, Na-K tartrate, and CuSO_4_ were purchased from Sigma-Aldrich–Fluka (Steinheim, Germany). Acrylamide, Bis-acrylamide, Silver stain kit, and Bradford reagent were purchased from Bio-Rad Laboratories Srl. (Milan, Italy). Pierce™ BCA Protein Assay Kit and complete RIPA lysis buffer (#89900, #87786) were obtained from Thermo Fisher Scientific (Waltham, MA, USA). Contents (1 mL): 25 mM Tris-HCl,150 mM NaCl, 1%NP-40, 1% sodium deoxycholate, 0.1% SDS, 1 mM AEBSF, 800 nM Aprotinin, 50 μM Bestatin, 15 μM E64, 20 μM Leupeptin, 10 μM Pepstatin A, and 5 mM EDTA.

## 5. Conclusions

In this study, we considered and compared a variety of methodologies and treatments for the quantification of proteins in LBFs. These approaches included (a) lysis buffer (homemade vs. RIPA), (b) protein assays (Lowry, BCA, and Bradford), (c) ultrasonic bath treatment, and (d) protein staining (silver staining and Coomassie Blue). On the basis of the comparative outcome, we suggest using the homemade lysis buffer, BCA, or alternatively the Lowry assay, ultrasonic bath treatment, and silver stain to maximize the extraction and characterization of protein for *A. lessonii*. This protocol might be suitable and extended to other benthic foraminiferal species, including the smaller ones and, more importantly, represents a baseline approach to be considered if *A. lessonii* is to be used as a biomarker.

## Figures and Tables

**Table 1:** Correlation coefficient and calibration curve equation for the considered protein assays (i.e., Lowry, BCA, and Bradford).

	Lowry	BCA	Bradford
**R^2^**	0.97	0.96	0.96
**Calibration curve equation**	y = 0.1829x − 0.03347	y = 0.1634x − 0.03222	y = 0.1607x − 0.004233

**Table 2:** Protein content (mg/mL) data per different treatments: lysis buffer: homemade (HM) and RIPA; assays: Lowry, BCA, and Bradford and ultrasonic bath treatment (US).

Ultrasound	Lysis	Assay		
	Buffers	Lowry	BCA	Bradford
**With**	**HM** [mg/mL]	0.85 ± 0.06	0.85 ± 0.03	0.37 ± 0.05
	**RIPA** [mg/mL]	0.51 ± 0.06	0.49 ± 0.05	0.27 ± 0.06
**Without**	**HM** [mg/mL]	0.61 ± 0.04	0.59 ± 0.03	0.31 ± 0.05
	**RIPA** [mg/mL]	0.39 ± 0.02	0.38 ± 0.01	0.23 ± 0.02

## Data Availability

Data is contained within the article.
